# Digitalisierung in der chirurgischen Weiterbildung: Status quo, Chancen und Herausforderungen

**DOI:** 10.1007/s00104-025-02285-0

**Published:** 2025-05-05

**Authors:** Carolina Vogel, Vera Bertsch, Mika F. Rollmann, Steven C. Herath, Tina Histing, Benedikt J. Braun

**Affiliations:** BG Klinik Tübingen, Klinik für Unfall- und Wiederherstellungschirurgie der Eberhard Karls Universität, Schnarrenbergstraße 95, 72076 Tübingen, Deutschland

**Keywords:** Chirurgische Weiterbildung, Digitalisierung, Digitale Transformation, Digitales Logbuch, Simulationen, Continuing surgical training, Digitalization, Digital transformation, Digital logbook, Simulations

## Abstract

**Hintergrund:**

Die digitale Transformation der Medizin stellt ein zentrales politisches Thema dar, welches auch die chirurgische Weiterbildung betrifft. Die bisherigen Bemühungen zur Umsetzung der Digitalisierung wurden von Ärztinnen und Ärzten als unzureichend wahrgenommen. Es besteht noch erhebliches Potenzial, die Chancen der Digitalisierung gezielt zur Verbesserung der chirurgischen Weiterbildung einzusetzen.

**Fragestellung:**

Wie bewerten Ärztinnen und Ärzte in (spezieller) chirurgischer Weiterbildung die Verfügbarkeit, Umsetzung sowie Chancen und Hindernisse der Digitalisierung in der chirurgischen Weiterbildung in Deutschland?

**Material und Methoden:**

Ärztinnen und Ärzte in (spezieller) chirurgischer Weiterbildung wurden über einen webbasierten Fragebogen zum Stand der Digitalisierung in der chirurgischen Weiterbildung befragt. Insgesamt wurden 313 Fragebögen ausgewertet.

**Ergebnisse:**

Die Mehrheit der Ärztinnen und Ärzte sieht großes Potenzial in der Digitalisierung zur Verbesserung der Weiterbildung. Digitale Weiterbildungen, Apps und Onlinedatenbanken werden regelmäßig genutzt. Demgegenüber verwendet lediglich ein Viertel der Befragten ein digitales Logbuch. Die Mehrheit der Befragten sieht in der Umsetzung der Digitalisierung zudem Verbesserungschancen bei der Vereinbarkeit von Familie und Beruf.

**Diskussion:**

Digitale Tools sowie die Nutzung von Augmented Reality (AR) und Virtual Reality (VR) werden als potenziell wertvoll zur Verbesserung der Weiterbildung angesehen. Bislang werden diese Möglichkeiten jedoch kaum genutzt. Die bislang unzureichende Implementierung digitaler Logbücher verdeutlicht die Notwendigkeit einer optimierten Integration dieser digitalen Lösungen. Eine umfassende digitale Transformation in der Chirurgie ist erforderlich, um die Effizienz im klinischen Alltag und die Qualität der Weiterbildung nachhaltig zu optimieren. Daher besteht sowohl hinsichtlich des Ausbaus der digitalen Infrastruktur in den Kliniken als auch bei der Integration theoretischer und praktischer digitaler Tools in die Weiterbildung dringender Handlungsbedarf.

**Graphic abstract:**

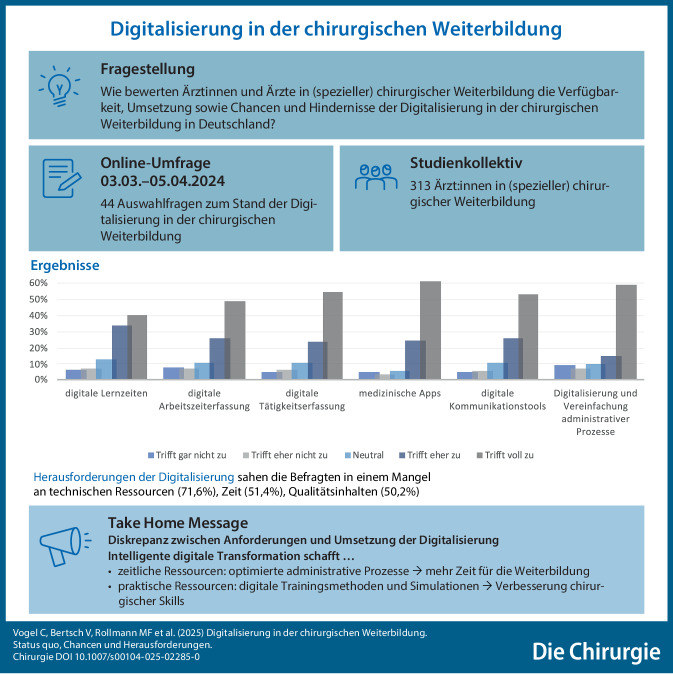

## Abstract (en)

**Background:**

The digital transformation of medicine is a key policy issue that also affects continuing surgical training. Previous efforts to implement digitalization were perceived as insufficient by doctors. There is still considerable potential to improve continuing surgical training by targeting the opportunities offered by digitalization.

**Research question:**

How do physicians in (special) surgical training assess the availability, implementation, opportunities and barriers of digitalization in continuing surgical training in Germany?

**Material and methods:**

Physicians in (special) surgical training were surveyed via a web-based questionnaire on the status of digitalization in continuing surgical training. A total of 313 responses were analyzed.

**Results:**

The majority of physicians see great potential in digitalization for improving continuing training. Digital training programs, apps and online databases are used regularly; however, only a quarter of respondents use a digital training logbook. The majority of respondents also see opportunities to improve the work-life balance through the implementation of digitalization.

**Conclusion:**

Digital tools as well as the use of augmented reality (AR) and virtual reality (VR), are seen as potentially valuable for improving continuing education but are scarcely used. The inadequate implementation of digital training logbooks highlights the need for optimized integration of these digital solutions. A comprehensive digital transformation in surgery is necessary to sustainably improve the efficiency of clinical routine practice and the quality of continuing education. Urgent action is therefore needed both in terms of expanding the digital infrastructure in hospitals and integrating theoretical and practical digital tools into continuing education.

## Hintergrund und Fragestellung

Die fortschreitende Digitalisierung stellt eine gesamtgesellschaftliche Herausforderung dar, welche alle Bereiche des Lebens betrifft. Auch das Gesundheitswesen ist von den damit einhergehenden Veränderungen betroffen. Die digitale Transformation, also die intelligente Nutzung digitaler Daten, wirkt sich im medizinischen Sektor sowohl auf die Krankenversorgung, Forschung, Lehre und damit auch auf die die chirurgische Weiterbildung aus [1, 2]. Derzeit stehen Politik, Hochschulen und medizinische Versorgungseinrichtungen vor großen Herausforderungen hinsichtlich der Finanzierung und Umsetzung der Digitalisierung [3–5]. Die Implementierung digitaler medizinischer Infrastruktur gewinnt somit zunehmend an Bedeutung und erfordert auch beim ärztlichen Personal eine Anwendungskompetenz [6–8]. Diese digitale Kompetenz erfordert u. a. die Fähigkeit, spezialisierte digitale Technologien und Systeme im Alltag effektiv zu nutzen. Hinsichtlich der chirurgischen Weiterbildung hat die Digitalisierung von Lehrinhalten besonders während der Corona-Pandemie eine rasante Entwicklung gezeigt. So können zum einen zunehmend theoretische Weiterbildungsinhalten digital einfach und zeitnah abgerufen werden, sodass sich diese im Rahmen eines Selbststudiums zeitökonomisch aneignen lassen [6, 9, 10]. Zum anderen bieten Simulationen in der operativen Weiterbildung bei zunehmender technischer Komplexität der Operationen sowie steigendem Zeit- und Kostendruck eine zusätzliche Trainingsoptionen zur Optimierung der Performance [11, 12]. Hinzu kommen spezielle Weiterbildungstools wie digitale Nachschlagewerke für das chirurgische Management, beispielsweise die Surgery-Reference-App der Arbeitsgemeinschaft für Osteosynthesefragen, smarte Bildschirme im Operationssaal oder intelligente Sprachsteuerungssysteme. Diese sollen dazu beitragen, den Arbeitsalltag sowie die Weiterbildung zu optimieren. Die Grenzen zwischen einer effizienten Bewältigung des digitalen, administrativen Klinikalltags und der daraus entstehenden Zeitersparnis und somit der Verbesserung der chirurgischen Weiterbildung sind fließend.

In der Realität wurde die Umsetzung der Digitalisierung von Ärztinnen und Ärzten bisher jedoch als insuffizient wahrgenommen und die möglichen Chancen kommen deshalb im Klinikalltag nur unzureichend zur Anwendung [9, 10]. Besonders im Kontext der ärztlichen Weiterbildung stehen vielerorts noch eine hohe Arbeitsbelastung mit einer Vielzahl an unzureichend digital transformierten administrativen Tätigkeiten, ökonomische Zwänge sowie die Einhaltung des Arbeitszeitgesetztes im Fokus und lassen teilweise wenig Zeit für die Weiterbildung [13].

Diese Umfrage untersucht die Verfügbarkeit, Umsetzung sowie Wahrnehmung der Chancen und Hindernisse der Digitalisierung in der chirurgischen Weiterbildung in Deutschland.

## Studiendesign und Untersuchungsmethoden

Die vorliegende Studie basiert auf einer anonymen Onlineumfrage, die im Zeitraum vom 03.03. bis 05.04.2024 durchgeführt wurde. Die Umfrage richtete sich an Ärztinnen und Ärzte in Weiterbildung sowie Fachärztinnen und Fachärzte in der speziellen chirurgischen Weiterbildung aller chirurgischer Fächer (Weiterbildungsassistenten, WBA). Die Umfrage wurde durch den Berufsverband der Deutschen Chirurgie (BDC) über den E‑Mail-Verteiler der Ärztinnen und Ärzte in Weiterbildung sowie der Fachärztinnen und Fachärzte geteilt. Des Weiteren wurde die Umfrage auf den Social-Media-Kanälen des Vereins Chirurginnen e. V. geteilt.

Im Rahmen der vorliegenden Untersuchung wurden die Verfügbarkeit, die Umsetzung sowie die Wahrnehmung der Chancen und Hindernisse der Digitalisierung in der chirurgischen Weiterbildung in Deutschland analysiert. Insgesamt umfasste der Fragebogen 44 Auswahlfragen mit teilweise freien Zusatzantworten sowie ein abschließendes Freitextfeld zur Übermittlung von Anregungen zum Thema. Im Rahmen der Umfrage wurden demografische Daten wie Geschlecht, Altersgruppe, beruflicher Tätigkeitsbereich (stationär oder ambulant), Arbeitszeitmodell, Wohnort, chirurgische Fachrichtung und Art der medizinischen Einrichtung erfasst. Darüber hinaus wurde der Status der Weiterbildung erfragt. Die Umfrage ermittelte die subjektive Einschätzung der digitalen Kompetenzen im privaten und beruflichen Kontext. Darüber hinaus wurde erfragt, wie die Vermittlung digitaler Kompetenzen im Rahmen des Medizinstudiums bewertet wird. Als digitale Kompetenzen wurden folgende Bereiche definiert: Informationstechnologie und Datenmanagement, Telemedizin und digitale Gesundheitsanwendungen, digitale Patientenversorgung und Simulationen, E‑Learning und Onlinebildungsressourcen, digitale Ethik und rechtliche Aspekte sowie interdisziplinäre Zusammenarbeit und Kommunikation.

Darüber hinaus wurden die Nutzung digitaler Tools im beruflichen Alltag sowie die Teilnahme an digitalen Weiterbildungsangeboten erfragt. Im Rahmen der Erhebung wurde zwischen verschiedenen digitalen Anwendungen wie Onlinedatenbanken, Nachschlagewerken, Podcasts und Onlineweiterbildungen differenziert. Des Weiteren wurde erhoben, ob die Teilnehmenden im Vorfeld eine Schulung in der Nutzung der jeweiligen Anwendungen erhalten hatten. Zusätzlich wurden Fragen zur Einschätzung der Potenziale digitaler Anwendungen für die Optimierung der Weiterbildung gestellt. Zudem wurden etwaige Hindernisse für die Nutzung digitaler Tools erfasst.

Als Umfragetool wurde die Software Google Formulare verwendet. Die Umfragedaten wurden deskriptiv ausgewertet. Die Analyse und Visualisierung der Umfragedaten erfolgte mit Excel (Microsoft® Excel® für Microsoft 365 MSO (Version 2406 Build 16.0.17726.20078), Redmond, WA, USA). Die Kategorisierung und Visualisierung der freien Antworten wurden händisch durchgeführt.

## Ergebnisse

### Demographische Daten

Insgesamt nahmen 313 Personen an der Umfrage teil. Die demographischen Daten sind in Tab. 1 dargestellt. Die Mehrheit der Befragten sind Frauen (70,3 % weiblich, 28,8 % männlich). 53 % der Teilnehmenden sind zwischen 30 und 39 Jahre alt. Rund drei Viertel arbeiten im stationären Sektor, ebenfalls knapp drei Viertel arbeiten in Vollzeit. Mehr als die Hälfte der Befragten lebt in Großstädten mit über 100.000 Einwohnern. Die häufigsten Fachrichtungen sind Viszeralchirurgie und Orthopädie/Unfallchirurgie (jeweils 31 %), gefolgt von Allgemeinchirurgie (14,7 %). Etwa die Hälfte (48,9 %) sind Fachärztinnen oder Fachärzte, die andere Hälfte (48,6 %) befindet sich in Weiterbildung. 27,2 % der Befragten arbeiten in einem Krankenhaus der Grund- und Regelversorgung, 16,3 % arbeiten in einer Universitätsklinik und nur 4,2 % sind in einer Praxis tätig.

**Tab. 1:** Demographische Daten der Teilnehmenden (*n* = 313)

	*n*	Anteil (%)
*Geschlecht*		
Männlich	90	28,8
Weiblich	220	70,3
Divers	1	0,3
Keine Antwort	2	0,6
*Altersgruppe*		
< 20 Jahre	1	0,3
20–29 Jahre	46	14,7
30–39 Jahre	166	53,0
40–49 Jahre	73	23,3
50–59 Jahre	11	3,5
60–69 Jahre	16	5,1
> 69 Jahre	0	0,0
*Tätigkeitssektor*		
Stationär	239	76,4
Ambulant	19	6,1
Gemischt	55	17,6
*Tätigkeitsregion*		
Ländliche Region (weniger als 10.000 Einwohnerinnen und Einwohner)	20	6,4
Kleinstadt und Mittelstadt (mehr als 10.000/weniger als 100.000 Einwohnerinnen und Einwohner)	124	39,6
Großstadt (mehr als 100.000 Einwohnerinnen und Einwohner)	169	54,0
*Karrierestufe*		
Studierende	1	0,3
Ärztliche Weiterbildung: 1. bis 2. Jahr	44	14,1
Ärztliche Weiterbildung: 3. bis 4. Jahr	29	9,3
Ärztliche Weiterbildung: > 4. Jahr	79	25,2
Fachärztin/Facharzt	153	48,9
Privatärztliche Tätigkeit	1	0,3
Oberärztin/-arzt	5	1,6
Chefärztin/-arzt	1	0,3
*Arbeitszeitmodell*		
Studium	1	0,3
Vollzeit	230	73,5
Teilzeit > 50 %	55	17,6
Teilzeit < 50 %	9	2,9
Elternzeit	13	4,2
Arbeitssuchend	5	1,6
*Versorgertyp*		
Praxis	13	4,2
Medizinisches Versorgungszentrum	9	2,9
Krankenhaus der Grund- und Regelversorgung	85	27,2
Krankenhaus der Zentralversorgung/Schwerpunktkrankenhäuser	74	23,6
Krankenhaus der Maximalversorgung	73	23,3
Universitätsklinik	51	16,3
Nichts davon ist zutreffend	8	2,6
*Fachdisziplin*		
Allgemeinchirurgie	46	14,7
Gefäßchirurgie	20	6,4
Herzchirurgie	9	2,9
Kinderchirurgie	10	3,2
Mund-Kiefer-Gesichtschirurgie	9	2,9
Neurochirurgie	1	0,3
Orthopädie und Unfallchirurgie	97	31,0
Plastische Chirurgie	11	3,5
Thoraxchirurgie	6	1,9
Viszeralchirurgie	97	31,0
Gynäkologie	1	0,3
Dermatologie	1	0,3
Zahnmedizin	3	1,0
Sonstige	2	0,6

### Chancen der Digitalisierung für die chirurgische Weiterbildung

**Abb. 1:**
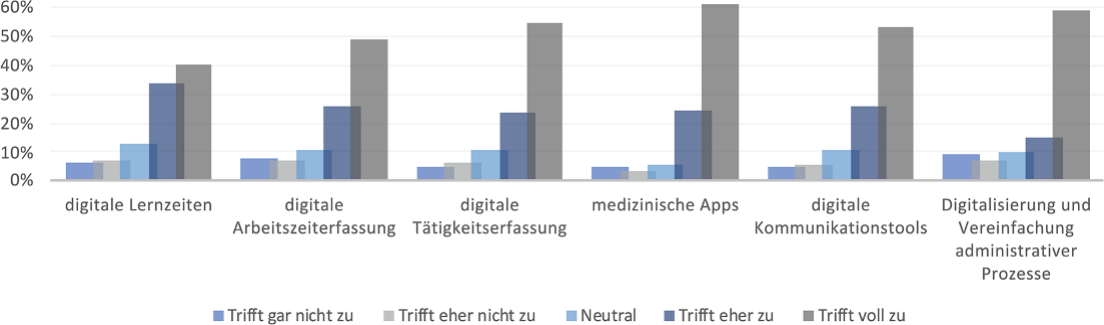
Digitalisierungsmaßnahmen mit potenziell positiver Auswirkung auf die Weiterbildung

Die Option digitaler Lernzeiten, die Digitalisierung der Arbeitszeit- und Tätigkeitserfassung, der Einsatz medizinischer Apps und Kommunikationsmittel sowie die Optimierung der digitalen Administration wird mehrheitlich positiv zur Verbesserung der Weiterbildung bewertet (Abb. 1).

Im Freitext wurden als gewünschte Digitalisierungsmaßnahmen die Verbesserung der intra- und interklinischen Infrastruktur sowie die Integration von künstlicher Intelligenz und Simulationen in die klinischen Abläufe und die Ausbildung genannt. Gefordert wurden beispielsweise der Einsatz von (KI-gestützten) Diktatprogrammen zur Erstellung von Arztbriefen sowie der Einsatz von OP-Simulationen zum Erlernen von Operationstechniken. Onlinekurse, Webinare und Apps mit klinischen Inhalten gehören ebenfalls zu den gewünschten Angeboten. Auch der Fernzugriff auf das Kliniksystem wird genannt.

**Abb. 2:**
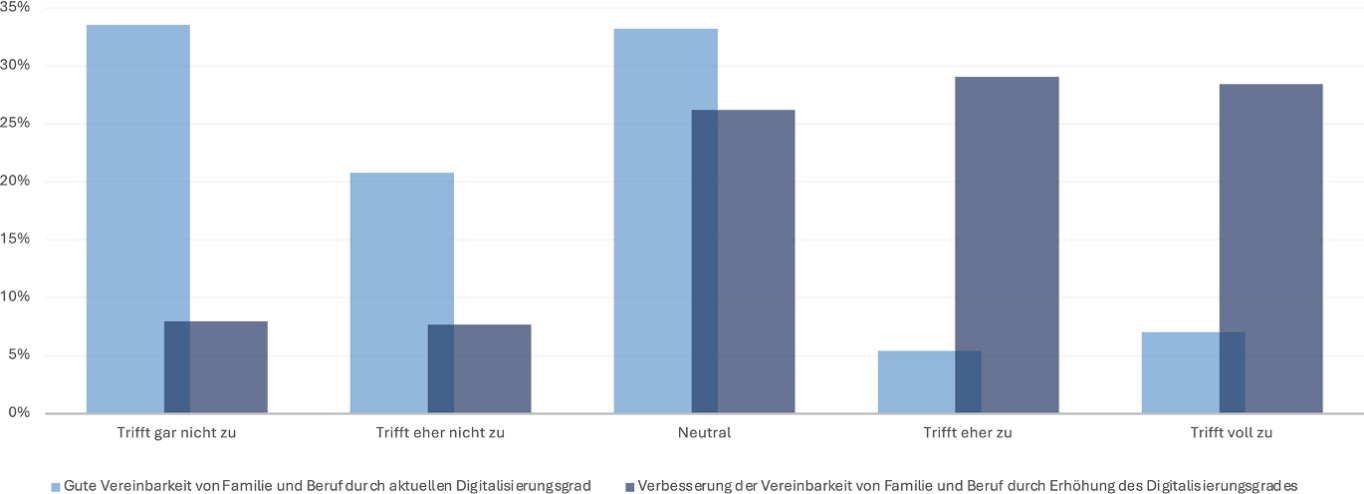
Verbesserung der Vereinbarkeit von Familie und Beruf durch Digitalisierung

In Bezug auf die Vereinbarkeit von Beruf und Familie zeigt sich, dass die Mehrheit der Befragten (54,3 %) keine Verbesserung durch den aktuellen Stand der Digitalisierung sieht. Allerdings sind 57,5 % der Befragten der Meinung, dass eine Verbesserung durch eine weitere Digitalisierung möglich ist (Abb. 2).

### Digitale Infrastruktur im Berufsalltag

**Abb. 3:**
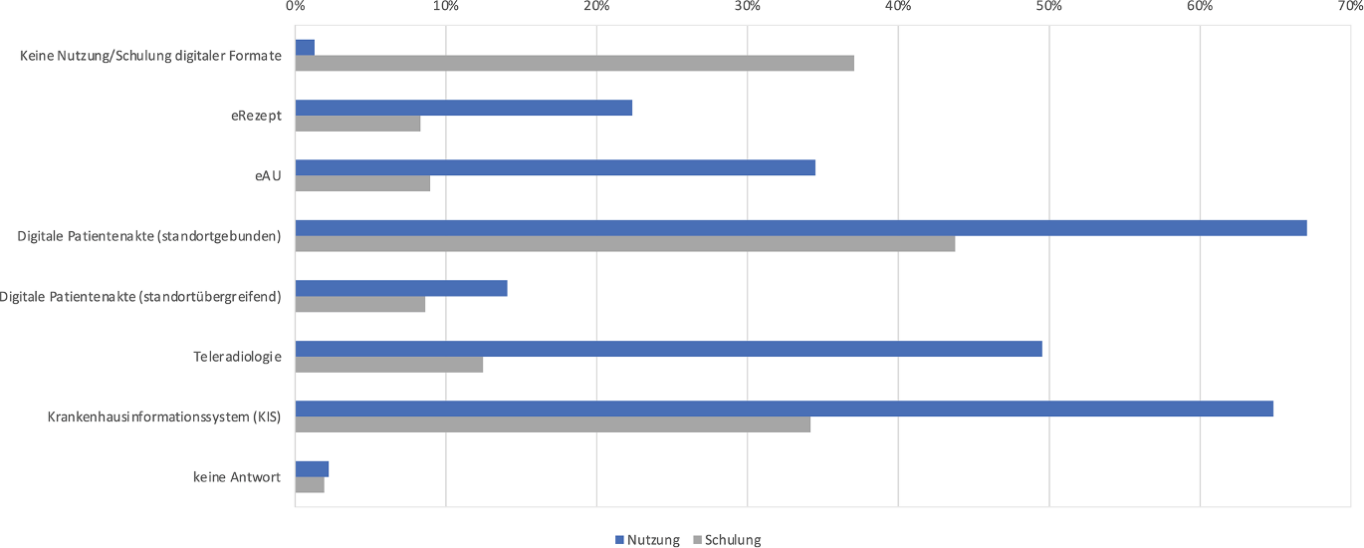
Nutzung und Schulung digitaler Anwendungen in medizinischen Versorgungseinrichtungen

Hinsichtlich der Nutzung digitaler Anwendungen im Berufsalltag (Abb. 3) geben 1,3 % der Befragten an, keinerlei digitale Anwendungen zu nutzen. Das elektronische Rezept wird von 22,4 % genutzt und die elektronische Arbeitsunfähigkeitsbescheinigung (eAU) von 34,5 % der Befragten. Die Mehrheit nutzt entweder standortgebundene oder standortübergreifende digitale Patientenakten, 64,9 % nutzen ein digitales Krankenhausinformationssystem. In diesem Kontext geben 37,1 % der Befragten an, in keiner von ihnen benutzten digitalen Anwendung geschult worden zu sein.

### Digitale Weiterbildungsformate

**Abb. 4:**
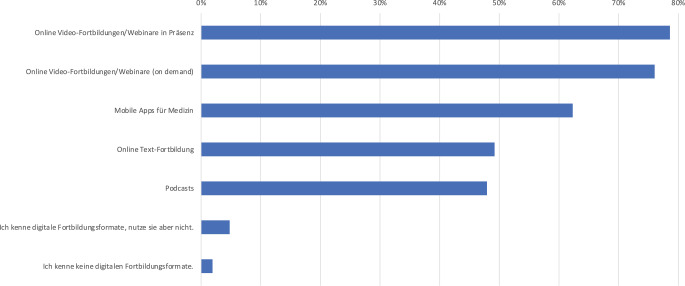
Nutzung digitaler Weiterbildungsformate

Digitale Weiterbildungsformate (Abb. 4) werden von der Mehrheit der Befragten eingesetzt. Dazu zählen Onlinedatenbanken und digitale Nachschlagewerke, die von 81,2 % der Befragten genutzt werden. Die Frequenz der Nutzung digitaler Nachschlagewerke wurde unabhängig von der Weiterbildungsstufe von mehr als zwei Drittel (78,6 %) als täglich oder wöchentlich angegeben (nicht dargestellt). Onlinefortbildungen werden von über 75 % der Teilnehmenden in Anspruch genommen. Zusätzlich nutzt etwa die Hälfte Podcasts zur Weiterbildung. Lediglich 6,7 % der Befragten geben an, keine digitalen Weiterbildungsformate zu kennen oder zu nutzen.

Dabei halten 54,3 % der Befragten digitale Formate für (eher) relevante Weiterbildungsmöglichkeiten, während 27,4 % der Befragten dem (eher) nicht zustimmen (nicht dargestellt).

**Abb. 5:**
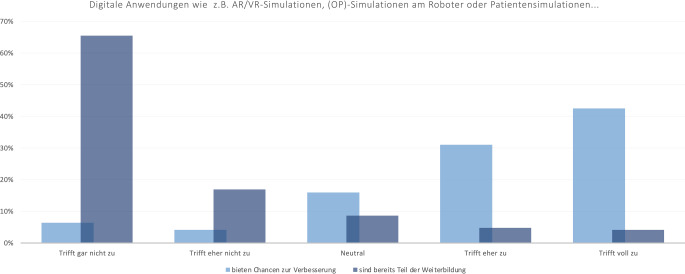
Einschätzung der Befragten zur aktuellen Verwendung digitaler Formate als Teil der Weiterbildung sowie der Chancen zur Verbesserung der Weiterbildung durch digitale Formate

Während 73,5 % der Befragten voll und eher zustimmen, dass digitale Anwendungen wie z. B. Augmented/Virtuell Reality, (OP-)Simulationen am Roboter oder Patientensimulationen Chancen zur Verbesserung der Weiterbildung bieten, geben 82,4 % an, dass diese aktuell nicht oder eher nicht Teil der Weiterbildung sind (Abb. 5).

### Digitale Kompetenz

Die Selbsteinschätzung der individuellen digitalen Kompetenz in „Anfänger“, „Fortgeschrittene“ und „Experte“ ist im privaten Bereich höher als im beruflichen (nicht dargestellt). Privat bewerten sich 72,8 % der Befragten als fortgeschrittene Nutzer und 11,8 % als Experten. Im beruflichen Umfeld sehen sich hingegen nur 58,8 % als Fortgeschritten und 5,8 % als Experten.

**Abb. 6:**
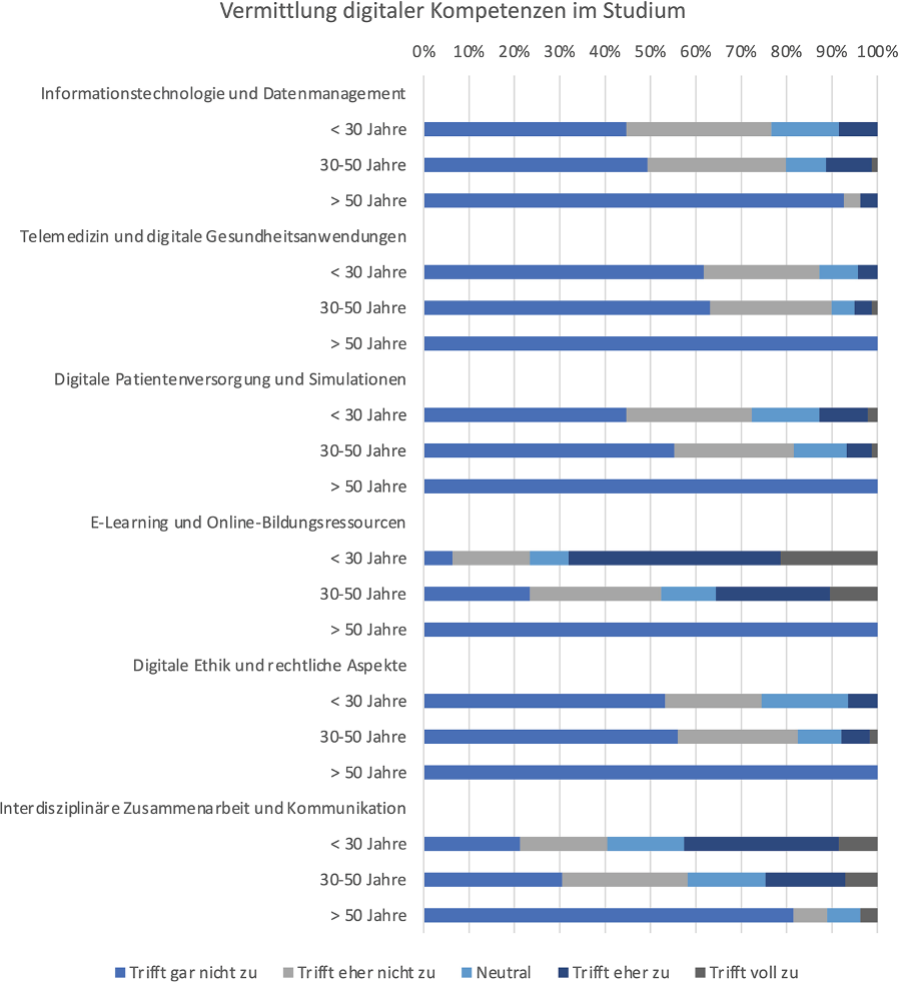
Einschätzung von im Studium vermittelten IT-Fähigkeiten

Die Vermittlung digitaler Kompetenzen im Medizinstudium als Vorbereitung auf die ärztliche Tätigkeit wurde mehrheitlich als unzureichend bewertet (Abb. 6). Lediglich die ausreichende Vermittlung von E‑Learning wird von rund zwei Dritteln der Befragten als zutreffend oder eher zutreffend eingeschätzt.

### Digitales Weiterbildungslogbuch

**Abb. 7:**
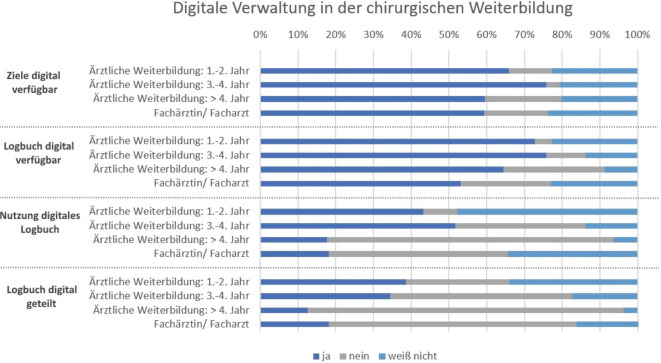
Nutzung des digitalen Weiterbildungslogbuchs

Im Rahmen der Umfrage wurde die Nutzung des digitalen Weiterbildungslogbuchs abgefragt (Abb. 7). Mehr als die Hälfte (60,7 %) der Befragten gibt an, dass für ihre Weiterbildung ein digitales Logbuch existiert, allerdings führen nur 24,6 % ein solches und nur 20,1 % teilen dieses auch digital mit den Weiterbildenden.

### Herausforderungen bei der Umsetzung der Digitalisierung

**Abb. 8:**
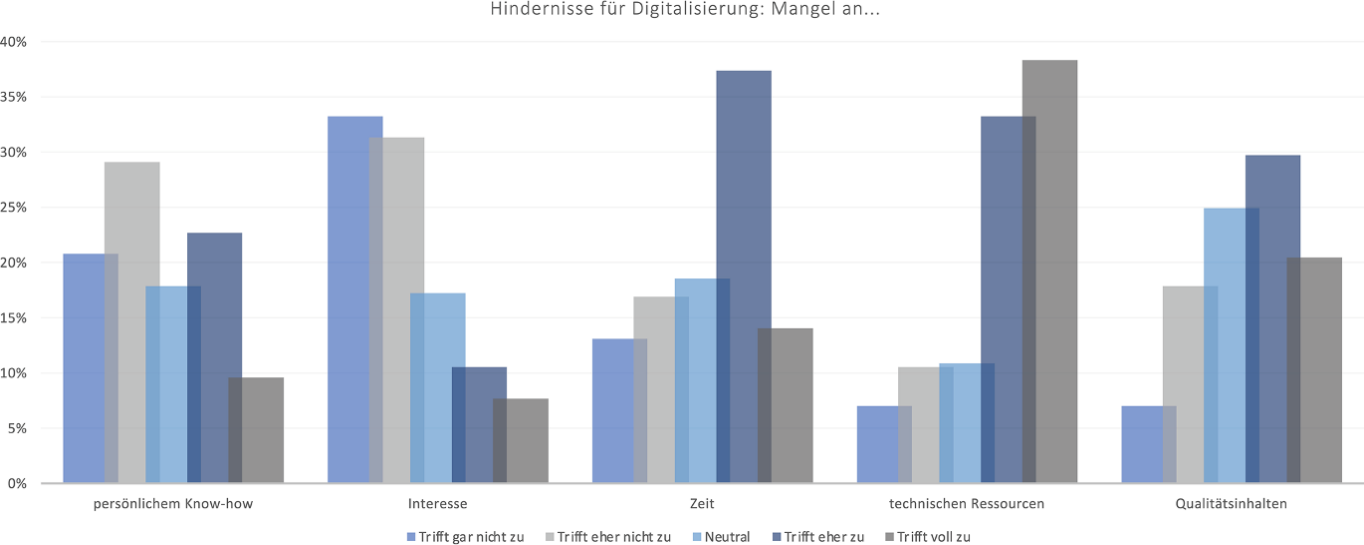
Hindernisse für Digitalisierung

Herausforderungen bei der Umsetzung der Digitalisierung sehen die Befragten hauptsächlich in einem Mangel an technischen Ressourcen (71,6 %), Zeit (51,4 %) und Qualitätsinhalten (50,2 %). Einen Mangel an persönlichem Know-how (49,8 %) oder Interesse (64,5 %) sieht die Mehrheit der Befragten eher nicht als Hindernis (Abb. 8).

## Diskussion

Die fortschreitende Digitalisierung im medizinischen Sektor ist unvermeidlich und umfasst auch die chirurgische Weiterbildung. Obwohl die digitale Transformation noch langsam voranschreitet und bei Finanzierung sowie Umsetzung deutliche Defizite bestehen, eröffnen sich perspektivisch wertvolle Chancen zur Verbesserung der klinischen Infrastruktur, zur Effizienzsteigerung administrativer Prozesse und damit potenziell mehr Zeit für die chirurgische Weiterbildung. Darüber hinaus entstehen zunehmend digitale Weiterbildungsformate sowohl theoretischer als auch praktischer Art, wie z. B. Simulationen, die eine wertvolle Ergänzung in der chirurgischen Ausbildung darstellen. Diese Umfrage beleuchtet die Einschätzung der Verfügbarkeit, Umsetzung und Wahrnehmung von Chancen und Hindernissen der Digitalisierung in der chirurgischen Weiterbildung in Deutschland.

Die Digitalisierung in der Chirurgie eröffnet für das medizinische Personal, die Kliniken und nicht zuletzt für die Patientinnen und Patienten erhebliche Optimierungspotenziale.

Die Möglichkeiten für die chirurgische Weiterbildung lassen sich jedoch nicht isoliert betrachten, sondern ergeben sich vielmehr aus einer Kombination verschiedener Faktoren. Dazu zählt zum einen die Optimierung der klinischen Infrastruktur. Diese umfasst den Abbau administrativer Tätigkeiten durch smarte Krankenhausinformationssysteme sowie die intelligente Dokumentation. Dies würde zu einer Steigerung der verfügbaren Zeit für die direkte Patientenversorgung sowie für die individuelle Weiterbildung führen. Eine Verbesserung der digitalen Infrastruktur wurde von der Mehrheit der Befragten dieser Umfrage zudem als Chance zur Verbesserung der Vereinbarkeit von Familie und Beruf bewertet.

Die Implementierung digitaler Wissensdatenbanken und neuer Technologien wie Simulationen oder telemedizinischer Infrastruktur kann zudem die Qualität der Weiterbildung erhöhen und letztlich zu einer besseren Patientenversorgung führen.

Digitale Formate wie medizinische Apps oder Wissensdatenbanken sind bereits im Berufsalltag der befragten Ärztinnen und Ärzten integriert. Die hohe Nutzungsrate und -frequenz bei der Mehrheit lässt auf eine große Akzeptanz digitaler Formate schließen. Die digitale Verfügbarkeit von Weiterbildungsinhalten bietet hohe Flexibilität und kommt einer komplexen Arbeits- und Dienstzeitrealität genauso entgegen wie der Vereinbarkeit von Privat- und Berufsleben. Zudem können Ärztinnen und Ärzte in (spezieller) chirurgischer Weiterbildung (WBA) theoretische Inhalte in ihrem individuellen Tempo abrufen und entsprechend verarbeiten. Auch die klassischen Kursformate erleben eine digitale Transformation. Eine Umfrage des Berufsverbands der Deutschen Chirurgie (BDC) aus dem Jahr 2022 zum Thema Fortbildungsformate zeigte, dass 30 % der Befragten Hybridveranstaltungen favorisieren [9]. Dieser Trend wird zunehmend in nationalen und internationalen digitalen Fortbildungsangeboten bedient [14]. Hinsichtlich der Umsetzungs- und Inhaltsqualität ist hierbei besonders die Schlüsselrolle der Fachgesellschaften zu betonen, die maßgeblich die Weiterbildung und die entsprechenden Curricula beeinflussen und somit die Digitalisierung von Fort- und Weiterbildungsangeboten nicht ausschließlich dem Industriesektor überlassen sollten. Es existieren hierzu bereits erfolgreiche Beispiele, wie das Weiterbildungscurriculum für arthroskopische Chirurgie der Gesellschaft für Arthroskopie und Gelenkchirurgie (AGA). Die AGA zeigt, dass digitale Angebote effektiv etabliert und evaluiert werden können [15–17]. Die Arbeitsgemeinschaft für Osteosynthesefragen (AO) setzt solche Hybridformate ebenfalls in ihren Kursen um, was eine effektive Mischung aus Präsenz- und Onlinelernen ermöglicht [17, 18].

Trotz der hohen Akzeptanz digitaler Weiterbildungsangebote berichten 82,4 % der Teilnehmenden dieser Umfrage, dass Formate wie Augmented und Virtual Reality (AR, VR), OP-Simulationen am Roboter oder Patientensimulationen aktuell wenig bis gar nicht in der Weiterbildung integriert sind – obwohl diese laut Umfrage ein großes Potenzial zur Verbesserung der Weiterbildungsqualität bieten würden. AR- und VR-Simulationen können ein grundlegendes praktisches Verständnis von Operationen vermitteln und die Lernkurve bei der Durchführung realer Operationen verkürzen [19–22]. Eine Metaanalyse in der Viszeralchirurgie zeigte, dass VR-Training die Qualität laparoskopischer Operationen durch Senkung der Fehlerraten und Verbesserung des Gewebehandlings signifikant steigern kann [12]. Die gewünschte breitere Implementierung von AR- und VR-Simulationen in der Weiterbildung erscheint dementsprechend sinnvoll, da diese Technologien vor allem das Potenzial besitzen, die praktische Ausbildung und die Patientensicherheit erheblich zu verbessern [17, 23].

Zudem eröffnet die Integration telemedizinischer Technologien, wie der Einsatz tragbarer Kameras und Headsets durch Chirurginnen und Chirurgen in (spezieller) Weiterbildung, neue Möglichkeiten. Sie bietet zum einen die Chance, Operationen virtuell, z. B. durch den chirurgischen Mentor zu unterstützen und zum anderen, Operationsvideos zur Weiterbildung bereitzustellen [24, 25]. Diese könnten standortunabhängig die Qualität der Weiterbildung steigern. WBA an kleineren Kliniken können so via Livevideos an komplexen Eingriffen teilnehmen, die an ihrem Standort nicht möglich sind.

Um digitale Anwendungen sowohl klinisch als auch im Kontext der individuellen Weiterbildung zu nutzen, benötigen Anwenderinnen und Anwender eine gewisse Kompetenz im Umgang mit dieser [26]. Die Befragten schätzen ihre individuelle digitale Kompetenz im privaten Sektor höher ein als im beruflichen Bereich. Dies könnte zum einen auf anwendungsfreundlichere Technologien und Innovationen im Privaten zurückzuführen sein, während diese im klinischen Alltag als unzureichend und veraltet wahrgenommenen werden [27]. Während der private Alltag von sprachgesteuerten Apps mithilfe künstlicher Intelligenz smart unterstützt werden kann, erscheint in der beruflichen Realität der Teilnehmenden das Faxgerät noch immer das Indikatorgerät für die defizitäre technische Entwicklung im Gesundheitswesen.

Die Diskrepanz der digitalen Kompetenz zwischen privatem und beruflichem Umfeld könnte auch Folge der noch unzureichenden Adressierung im Studium sein [14, 28]. Aufgrund der stetigen Weiterentwicklung und teilweise auch Komplexität digitaler Innovationen kann die universitäre Lehre im Medizinstudium die Vermittlung digitaler Kompetenzen jedoch nicht vollumfänglich abbilden. Hierzu haben sich bereits alternative Studiengänge, wie z. B. die Medizininformatik entwickelt. Ziel sollte es dennoch sein, grundlegende Kompetenzen hinsichtlich des Umganges mit medizinischen Daten, Grundlagen der Telemedizin und den digitalen Aspekten der Patientenversorgung zu vermitteln. Dies macht sich auch im Referentenentwurf des Bundesministeriums für Gesundheit zur Neuregelung des Medizinstudiums bemerkbar, hier wird u. a. auch die Vermittlung von Kompetenzen bei digitalen Technologien gefordert. Auch sollen teilweise analoge durch digitale Formate ergänzt werden [29]. Die Befragten berichten zudem über ein Schulungsdefizit von Arbeitgebern bei digitalen klinischen Anwendungen. Ein strukturiertes digitales Onboarding-Programm für Ärztinnen und Ärzte, das spezifisches und fallbasiertes Training digitaler Anwendungen sowie flexible Beratungsangebote durch IT-Fachpersonal umfasst, könnte diese Lücken schließen. Dies erfordert jedoch eine funktionierende Hardware, intelligente Softwarelösungen und schließlich eine entsprechende personelle Ausstattung von IT-Abteilungen [27].

Im Kontext der Digitalisierung des Gesundheitssystems wurde 2018 durch den Deutschen Ärztetag sowohl ein strukturiertes Weiterbildungsprogrammes als auch die Einführung eines digitalen Logbuchs für Ärztinnen und Ärzte in Weiterbildung beschlossen. Dieses soll die Planung der Weiterbildung sowie eine kontinuierliche Dokumentation und Bewertung des individuellen Weiterbildungsstandes gewährleisten [30]. Sechs Jahre nach dem Beschluss geben in dieser Umfrage nur ein Viertel der Befragten an, ein solches zu nutzen. Damit liegen chirurgische WBA unter dem Bundesdurchschnitt, da eine Umfrage des Ärztetages 2023 ergab, dass immerhin 42 % der gesamten Ärztinnen und Ärzte in Weiterbildung das digitale Logbuch nutzen würden [31]. Ein Hindernis für die Nutzung könnte darin bestehen, dass die Popularität des digitalen Logbuches sehr gering erscheint, rund 20 % der Befragten kennen die (digital verfügbaren) Ziele ihrer Weiterbildung nicht. Der Einsatz des digitalen Logbuchs bietet jedoch neben der individuellen Weiterbildungsdokumentation u. a. Chancen für eine bessere Vergleichbarkeit der Weiterbildungen, da Anforderungen im Weiterbildungskatalog digital zwischen den Bundesländern abgeglichen werden können [32]. Das digitale Logbuch birgt großes Potenzial, die Transparenz und Vergleichbarkeit der ärztlichen Weiterbildung zu verbessern. Zudem lässt sich durch das implementierte strukturierte Weiterbildungsprogramm die Qualität der Weiterbildung langfristig steigern. Die flächendeckende Umsetzung sollte durch eine benutzerfreundliche und unbürokratische Anwendung sowohl für Weiterbildungsassistenten als auch für Weiterbilder gezielt gefördert werden.

Als wesentliche Hindernisse für die Digitalisierung wurden von den Befragten vor allem der Mangel an technischen Ressourcen sowie ein Mangel an Zeit genannt. Im Gesamtkontext der digitalen Transformation in Deutschland könnte dies auch auf eine Umsetzungsschwäche digitaler Projekte im Gesundheitssektor zurückzuführen sein, welche durch beispielsweise hohe Datenschutzanforderungen und teilweise inkonsistente Planung entsteht [33]. Ein weiteres Hindernis könnte in dem begrenzt vorhandenen digitalen Know-how liegen, welches bei rund 40 % der Befragten vorhanden ist. Dadurch wird eine effektive Nutzung digitaler Systeme erschwert. Hohe Datenschutzanforderungen und die Umsetzungsschwäche digitaler Projekte führen in Deutschland oft zu komplexen und langwierigen Implementierungsprozessen. Die Umfrage zeigt jedoch, dass das Interesse an Digitalisierung im medizinischen Sektor bei rund 60 % durchaus vorhanden ist. Um die Digitalisierung weiter voranzutreiben, sind gezielte Investitionen in die technische Infrastruktur sowie Schulungen zur digitalen Kompetenz erforderlich, um die Potenziale der digitalen Transformation besser ausschöpfen zu können.

## Limitationen

Als Limitation der Studie ist zu berücksichtigen, dass die teilnehmende Gruppe demografisch von der Gesamtheit der chirurgisch tätigen Ärzte und Ärztinnen abweicht.

Die Ergebnisse unserer Umfrage zeigen deutliche Abweichungen im Vergleich zur Gesamtärzteschaft. Im Jahr 2022 waren 30 % der Ärztinnen und Ärzte in Deutschland zwischen 40 und 49 bzw. 50 und 59 Jahre alt. Der Schwerpunkt dieser Umfrage liegt mit 53 % in der Altersgruppe 30 bis 39 Jahre, gefolgt von 40 bis 49 Jahren (23 %). Des Weiteren waren 76 % der Befragten stationär und nur 6 % ambulant tätig. Im Vergleich dazu sind 47 % bzw. 25 % der in Deutschland tätigen Ärztinnen und Ärzte stationär bzw. ambulant tätig [34]. Zudem sind in der vorliegenden Studie ca. 70 % der Befragten weiblich, während von allen in Deutschland chirurgisch Tätigen die Mehrheit mit ca. 78 % männlich ist [34]. Es kann somit möglicherweise nicht uneingeschränkt auf die in der Realität bestehende Gruppe rückgeschlossen werden. Des Weiteren wurde die Einladung zwar ausschließlich an Ärztinnen und Ärzte versandt, eine Prüfung derjenigen, die die Umfrage letztendlich ausgefüllt haben, wurde jedoch nicht durchgeführt. Mehrfachbeteiligungen können nicht ausgeschlossen werden. Der Einsatz eines Onlinefragebogens könnte technikaffine Ärztinnen und Ärzte stärker zur Teilnahme motiviert haben, was sowohl zu einer Verzerrung der Altersstruktur als auch der vertretenen Fachdisziplinen geführt haben könnte (Selektionsbias).

## Schlussfolgerung/Fazit für die Praxis

Die Ergebnisse der Umfrage zeigen, dass die Digitalisierung in der Chirurgie zwar zunehmend akzeptiert wird, jedoch durch strukturelle und organisatorische Hindernisse noch deutlich eingeschränkt ist. Dabei wird deutlich, dass eine Verbesserung der klinischen Infrastruktur sowohl positive Auswirkungen auf die Patientenversorgung als auch auf die chirurgische Weiterbildung hat. Beispielsweise bedeutet die intelligente Entlastung des ärztlichen Personals von administrativen Aufgaben mehr Zeit für die Patientenversorgung und damit auch mehr Zeit für die individuelle Weiterbildung in einem praktischen Fach. Zusätzlich eröffnen digitale Formate wie Simulationen und telemedizinische Technologien wertvolle neue Möglichkeiten zur Ergänzung der chirurgischen Weiterbildung, die jedoch möglicherweise aufgrund mangelnder Ressourcen und Zeit häufig nur unzureichend genutzt werden. Um die Digitalisierung wirksam voranzutreiben, sind gezielte Investitionen in die technische Infrastruktur und die Entwicklung benutzerfreundlicher digitaler Tools erforderlich. Ziel ist es, die Qualität der chirurgischen Weiterbildung und letztlich die Patientenversorgung nachhaltig zu verbessern.

## Data Availability

Die erhobenen Datensätze können auf begründete Anfrage in anonymisierter Form beim korrespondierenden Autor angefordert werden. Die Daten befinden sich auf einem Datenspeicher am BG Klinikum Tübingen.
